# Control of Temperature on Microbial Community Structure in Hot Springs of the Tibetan Plateau

**DOI:** 10.1371/journal.pone.0062901

**Published:** 2013-05-07

**Authors:** Shang Wang, Weiguo Hou, Hailiang Dong, Hongchen Jiang, Liuqin Huang, Geng Wu, Chuanlun Zhang, Zhaoqi Song, Yong Zhang, Huilei Ren, Jing Zhang, Li Zhang

**Affiliations:** 1 State Key Laboratory of Biogeology and Environmental Geology & School of the Earth Sciences and Resources, China University of Geosciences, Beijing, China; 2 Department of Geology and Environmental Earth Science, Miami University, Oxford, Ohio, United States of America; 3 State Key Laboratory of Biogeology and Environmental Geology, China University of Geosciences, Wuhan, China; 4 State Key Laboratory of Marine Geology, School of Ocean of Earth Sciences, Tongji University, Shanghai, China; 5 Department of Marine Sciences, the University of Georgia, Athens, Georgia, United States of America; 6 Laboratory for Conservation and Utilization of Bio-Resources, Yunnan Institute of Microbiology, Yunnan University, Kunming, China; University of Waterloo, Canada

## Abstract

The Tibetan Plateau in Northwest China hosts a number of hot springs that represent a biodiversity hotspot for thermophiles, yet their diversity and relationship to environmental conditions are poorly explored in these habitats. In this study we investigated microbial diversity and community composition in 13 Tibetan hot springs with a wide range of temperatures (22.1–75°C) and other geochemical conditions by using the 16S rRNA gene pyrosequencing approach. *Bacteria* (10^8^–10^11^ copy/g; 42 bacterial phyla) in Tibetan hot springs were more abundant and far more diverse than *Archaea* (10^7^–10^10^ copy/g; 5 archaeal phyla). The dominant bacterial phyla systematically varied with temperature. Moderate temperatures (75–66°C) favored Aquificae, GAL35, and novel *Bacteria*, whereas low temperatures (60–22.1°C) selected for Deinococcus-Thermus, Cyanobacteria, and Chloroflexi. The relative abundance of Aquificae was correlated positively with temperature, but the abundances of Deinococcus-Thermus, Cyanobacteria, and Chloroflexi were negatively correlated with temperature. Cyanobacteria and Chloroflexi were abundant in Tibetan hot springs and their abundances were positively correlated at low temperatures (55–43°C) but negatively correlated at moderate temperatures (75–55°C). These correlation patterns suggest a complex physiological relationship between these two phyla. Most archaeal sequences were related to Crenarchaeota with only a few related to Euryarchaeota and Thaumarchaeota. Despite the fact that microbial composition in Tibetan hot springs was strongly shaped by temperature, microbial diversity (richness, evenness and Shannon diversity) was not significantly correlated with temperature change. The results of this study expand our current understanding of microbial ecology in Tibetan hot springs and provide a basis for a global comparison.

## Introduction

Microbial community composition and diversity in terrestrial hot springs have been extensively studied, especially in Yellowstone National Park (YNP) [1]–[6], Japan [7]–[11], Great Basin [12]–[14], Iceland [15], [16], Thailand [17]–[19], and Russia [20]–[23]. Most of these phylogenetic studies have used 16S rRNA clone libraries in combination with cultivation methods. The results of these studies [2], [7], [11], [14], [16], [19], [22], [24]–[26] have expanded our knowledge of thermophilic microorganisms that commonly inhabit thermal springs. Thermophilic or hyperthermophilic *Bacteria* are commonly present in high-temperature hot springs (>75°C) [19], [27], [28] such as the phyla Aquificae, Deinococcus-Thermus, Thermodesulfobacteria, Thermotogae, and some thermophilic members within Proteobacteria and Firmicutes. When temperature is suitable for photosynthesis (<75°C), moderately thermophilic and mesophilic *Bacteria* are important members in terrestrial thermal springs, such as Cyanobacteria, Chloroflexi and Proteobacteria [10], [29], [30]. In addition to *Bacteria*, archaeal phyla Crenarchaeota, Euryarchaeota, and Thaumarchaeota are also commonly detected in geothermal systems [31], [32].

To better understand microbial ecology and functions in geothermal niches, an increasing number of studies have tried to establish the linkage between microbial community composition/diversity and physicochemical conditions such as temperature, pH, and water chemistry [7], [11], [16], [19], [30], [33], [34]. Among these environmental conditions, the effect of temperature on microbial community structure has received much attention. A previous study [7] reported that microbial communities in streamers with a temperature over 66°C was dominated by Aquificae, Thermodesulfobacteria, *Thermus*, and Crenarchaeota, but in microbial mats with a cooler temperature (<60°C), *Synechococcus*, *Chloroflexaceae*, *Rhodothermus* and Armatimonadetes (previous candidate division OP10) became predominant. A recent study [11] reported similar results showing that *Sulfurihydrogenibium* of the phylum Aquificae was the dominant component in microbial mats with temperatures of 75–67°C, but anoxygenic phototrophic *Chloroflexus* was the major group at lower temperatures (66–60°C). When temperature decreased below 60–58°C, oxygenic phototrophs *Thermosynechococcus*/*Synechococcus* became important.

Other studies [5], [16], [19] have focused on the relationship between microbial diversity and temperature; in general, microbial diversity was inversely correlated with temperature, or temperature was a key factor in controlling microbial diversity in hot springs. Recent studies [5], [14], [28] have employed high-throughput sequencing techniques to investigate microbial diversity and community composition in hot springs in relationship to temperature and other environmental conditions. In general, microbial diversity decreases with increased temperature and resemblance of microbial community composition between hot springs decays with increased difference in temperature [5], [14]. Likewise, our previous study [28] investigated hot springs from two geothermal regions of Tengchong, China, with a temperature range of 55.1–93.6°C and a pH range of 2.46 to 9.39, and found that temperature was a key factor in controlling microbial community distribution. Despite these recent studies, more effort is still needed to examine the effect of temperature on microbial abundance, diversity, and community composition at moderate temperatures. To date, no comprehensive studies have been performed for Tibetan hot springs using high throughput sequencing method and it is not clear if the relationship of microbial community composition, abundance, and diversity to physicochemical conditions in these springs differs from those at other terrestrial springs.

The Tibetan Plateau in NW China is the largest, highest, and also one of the youngest plateaus on Earth, and it is usually referred to as the “Roof of the World”. The plateau hosts a number of hot springs at high-elevations (>4500 m above sea level). Compared to a large number of studies on terrestrial hot springs at lower elevations, only a few hydrological and microbiological studies have been performed on high-elevation Tibetan hot springs [26], [29], [35]–[37]. Near-neutral to slightly alkaline pH, moderate temperatures, and low sulfide concentrations are some common features of Tibetan hot springs [26], [29], [36], [37]. In these springs, diverse microbial phyla in mats/streamers/sediments have been documented [26], [29], [36], [37], including archaeal phyla Crenarchaeota, Euryarchaeota, and Thaumarchaeota, and various bacterial phyla such as Cyanobacteria, Chloroflexi, Chlorobi, Proteobacteria, Firmicutes, and Deinococcus-Thermus. In particular, Cyanobacteria and Chloroflexi are dominant components in Tibetan springs with temperature lower than 70°C [26], [29], suggesting that photosynthesis is a major pathway of primary production in these springs. However, the relationship between these two phyla is still unclear in hot spring environments. The initial view of the strict cross-feeding relationship between these two phyla [38] has been questioned by later studies, which show that filamentous *Chloroflexi* are capable of growing photoautotrophically [39]–[45] and thus possibly competing against Cyanobacteria for limiting nutrients or physical space when they co-occur in the same environment [5]. This competing relationship has been supported by a recent study of photosynthetic activity in a hypersaline microbial mat [44]. In oxic layer, anoxygenic phototrophs (*Chloroflexus*-like bacteria) outcompete oxygenic phototrophs (Cyanobacteria-like) for inorganic carbon under the condition of full-light illumination. Clearly further research is needed to study the relationship between filamentous *Chloroflexi* and Cyanobacteria in an environment where both are present along an environmental gradient such as temperature. In addition, our understanding of the relationship between the overall microbial community composition and environmental parameters in Tibetan hot springs is still incomplete, largely due to the lack of application of high-throughput sequencing method to these springs and few environmental parameters measured.

The objective of this research was to study the microbial community composition and diversity in hot springs of the Tibetan Plateau across a wide range of temperatures. An integrated approach was employed including comprehensive geochemical analyses and high-throughput pyrosequencing. The relationship between Cyanobacteria and Chloroflexi was explored and discussed because both phyla were abundant in Tibetan hot springs. The results of this study expand our current understanding of microbial ecology in high-elevation Tibetan hot springs.

## Materials and Methods

### Site Description

The India-Asian continental collision resulted in a series of geological processes in Southwestern Asia, including crustal thickening, upwelling of the upper mantle, and the uplift of the Tibetan Plateau. All these processes led to the formation of near south-north striking rift basins on the Tibetan Plateau, where geothermal activities are abundant. The Naqu - Yangbajing - Yadong normal fault is one of the representative faults on the plateau and it stretches through Naqu County of Tibet [35]. Geothermal springs are abundant along this fault.

### Field Measurements and Sample Collection

**Figure 1:**
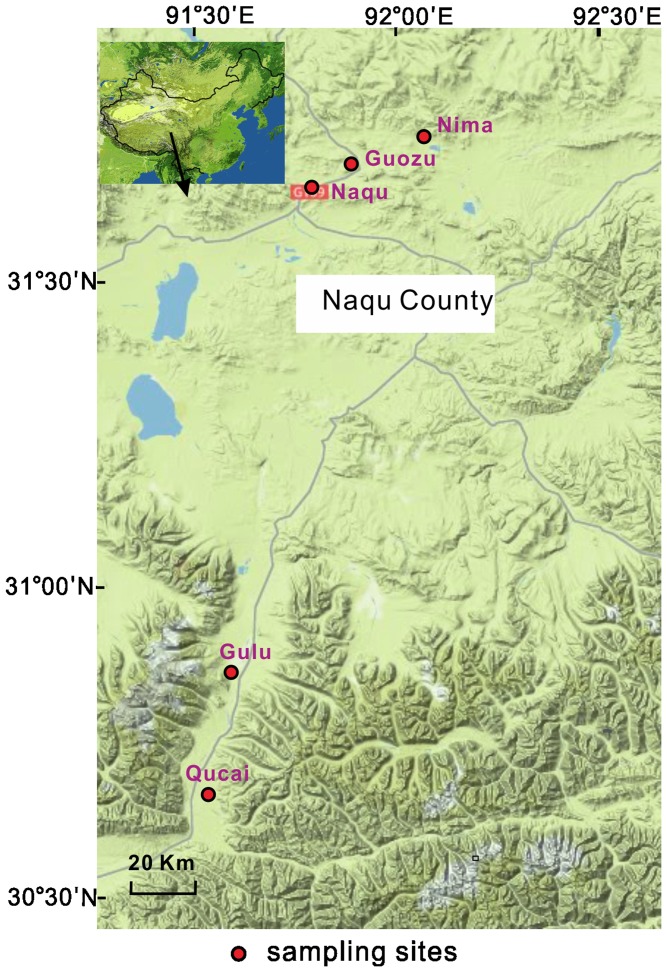
Geographic map showing sampling locations. The red dots along the highway represent the sampling areas in Naqu County of Tibet, NW China.

A total of thirteen hot springs in five areas of the Tibetan Plateau, i.e., Nima (NM), Gulu (GL), Naqu (NQ), Guozu (GZ), and Qucai (QC) in Naqu County, were selected for field measurements and sample collection (Figure 1). The springs in Naqu County have been categorized as Na-Cl and Na-HCO_3_ type [35]. At each hot spring, water temperature, pH, and total dissolved solids (TDS) were measured using a temperature probe (YSI, Yellow Springs, OH, USA) and Hach meter (equipped with pH and TDS probes), respectively. After field measurements, sediment samples were collected into 50-mL falcon tubes and immediately frozen in liquid nitrogen. Some small and shallow springs (such as GL20) contained microbial mats in spring water, therefore the collected samples from these springs likely contained a certain amount of mat material. Two adjacent soil samples, one from Gulu (GLs) and the other from Nima (NMs), were collected for the purpose of assessing possible contamination of spring microbial communities by nearby soil microbes. The samples were kept in liquid nitrogen in the field and on dry ice during transportation. Once in laboratory, they were stored at −80°C until further analysis.

### Geochemical Analyses

Pore water from the sediment samples was separated by centrifugation and cations and anions were measured by direct current plasma optical emission spectrometry (DCP-OES, Beckman, USA) and Dionex ion chromatography (AS14A column, with 10 µM Na_2_CO_3_/NaHCO_3_ as an eluent, Dionex, USA), respectively. Total nitrogen (TN) and total organic carbon (TOC) of the sediment samples were determined using an NC 2100 Elemental Analyzer after fumigating sediment with HCl to remove carbonates. Quantitative XRD analysis was performed to identify the mineralogy using the procedures described in a previous study [37]. Briefly, one gram of ground sample was mixed with 0.25 g of an internal standard (corundum). This mixture along with 4 ml ethanol and 2 ceramic beads was vortexed (Vortex Genie, Scientific Ind. Inc., USA) for 10 min. After drying at 65°C overnight, 600 µL of DuPont Vertrel XF (Miller-Stephenson, Sylmar, CA, USA) was added to the mixture followed by drying at room temperature for another 10 min. In order to avoid the orientation effect of mineral particles, the powder was side-packed into a holder. Samples were X-ray scanned from 2 to 70 degree two theta with Cu K-alpha radiation (40 kV, 35 mA), a 0.02 degree step size, and a count time of 5 seconds per step. The XRD data were analyzed quantitatively and converted into weight percent using the RockJock computer program (detection limit 0.1%) [46].

### DNA Extraction, Polymerase Chain Reaction (PCR) Amplification, and 454 Pyrosequencing

Fifteen samples (13 hot springs and 2 adjacent soils) were subjected to DNA extraction using the FastDNA Spin Kit for Soil (MP Biomedicals, OH, USA) according to the manufacturer’s protocol. Duplicate extractions were performed for each sample and each extraction was from ∼0.5 g wet sediments. DNA aliquots from two replicate extractions were pooled, and the DNA quality and concentrations were assessed based on the absorbance ratios of 260/280 and 230/280 using a NanoDrop ND-1000 Spectrophotometer (NanoDrop Technology, DE, USA).

For all the fifteen samples, the V4–V8 variable region of the bacterial and archaeal 16S rRNA genes was amplified with the forward primer 515F-M (5′-GTGYCAGCMGCCGCGGTAA-3′) [28] and the reverse primer 1391R (5′-GACGGGCGGTGWGTRCA-3′) [47]. Both forward and reverse primers for each sample were bar-coded with a unique 8-bp tag at the 5′-end. Between each barcode and the primer sequence, a linker sequence was inserted (‘TC’ and ‘CA’ for the forward and reverse primers, respectively). A typical PCR reaction (25 µL in volume) consisted of the following reagents: 2.5 µL 10×PCR buffer (TaKaRa, Dalian, China), 2.0 µL dNTP (TaKaRa, Dalian, China), 1.0 µL of each primer (10 µM), 1.0 µL bovine serum albumin (TaKaRa, Dalian, China), 0.25 µL of rTaq DNA polymerase (TaKaRa, Dalian, China), and 1.0 µL of genomic DNA (∼20–50 ng) as a template. PCR conditions were as follows: an initial denaturation step at 95°C for 5 min, 35 cycles of denaturing at 94°C for 30 s, annealing at 54°C for 30 s, extension at 72°C for 1 min, and a final extension step at 72°C for 10 min. To obtain enough PCR product for sequencing, five PCRs were performed for each sample and the amplicons were pooled. The products were confirmed by agarose gel electrophoresis, purified with the Agarose Gel DNA Purification Kit Ver.2.0 (TaKaRa, Dalian, China). The purity was assessed with a NanoDrop ND-1000 Spectrophotometer (NanoDrop Technologies, Wilmington, DE, USA). Amplicons of all samples were mixed with equimolar concentrations for 454 pyrosequencing, which was performed with a second generation 454 GS FLX Titanium technology (454 Life Sciences, Branford, CT, USA) at the Chinese National Human Genome Center in Shanghai.

### Quantitative PCR (qPCR) for Bacteria, Archaea, Chloroflexi, and Cyanobacteria 16S rRNA Genes

The abundances of total *Bacteria*, *Archaea*, Chloroflexi, and Cyanobacteria were quantified with qPCR in polypropylene 96-well plates on an ABI 7500 fast real-time PCR sequence detection system (Applied Biosystems, USA). Specific primers for these four groups were used (Table 1). Each 20 µL reaction contained the following ingredients: 10 µL of the Power SYBR Green PCR Master Mix (Applied Biosystems, USA), 0.5 µL of each primer (10 µM; Sangon), 1.0 µL of bovine serum albumin (Takara), 7 µL of H_2_O, and 1.0 µL of template DNA (∼20–50 ng). Plasmid standards were serially diluted from 10^9^ to 10^2^. The qPCR cycling conditions were 30 s at 95^o^C for initial denaturing, followed by 40 cycles of denaturing at 95^o^C for 30 s, annealing at a specific temperature for a given microbial group (Table 1) for 30 s, and extension at 72^o^C for 1 min. After the amplification, a dissociation step was added to check for the specificity of the amplicons and for any experimental artifacts such as the formation of primer-dimers. The annealing temperature was experimentally optimized to maximize the specificity of the PCR amplicons. When the run was complete, the amplicons were run on a 1.2% agarose gel to further confirm the specificity.

**Table 1:** Primer sets used for qPCR.

Targeted group	Primer sets	Sequence(5′-3′)	Annealing T(^o^C)	Reference
*Bacteria*	518F	CCAGCAGCCGCGGTAAT	58	[73]
	786R	CTACCAGGGTATCTAATC		[74]
*Archaea*	344F	ACGGGGTGCAGCAGGCGCGA	56	[75]
	518R	ATTACCGCGGCTGCTGG		[73]
Chloroflexi	CCR-344-F	ACGGGAGGCAGCAGCAAG	55	[76]
	CCR-1338-R	ACGCGGTTACTAGCAACT		
Cyanobacteria	CYA359F	GGGGAATYTTCCGCAATGGG	55	[77]
	CYA781R	GACTACWGGGGTATCTAATCCCWTT		

### Sequence Processing

Pyrosequencing errors were minimized by removing reads with either low quality scores (<25), or ambiguous bases/errors in barcodes, or more than two mismatches in primer sequences. In addition, those reads with homopolymers more than 6 or length shorter than 120 bp were discarded. All the sequencing-error checking steps were performed in Mothur [48]. The following steps were subsequently completed by using the QIIME software package. To make meaningful comparisons of microbial diversity across multiple samples and also to reduce sequence processing time all sequences that passed quality filtering were trimmed to 239 bp according to the quality score curve (not shown). OTUs were defined at the similarity levels of 80%, 90%, 95%, and 97% using UCLUST [49]. These OTU levels approximately correspond to the phylum, order, genus, and species level, respectively [50]. The most abundant sequence from each OTU was selected as a representative, and the representatives from all OTUs were then aligned with the PyNAST method [51]. With the downloaded database from GreenGenes (http://greengenes.lbl.gov; 16S.gold.NAST_ALIGNED.fasta and training set gg_97_otus_4feb2011.fasta), the aligned sequences were sent through ChimeraSlayer [52] and BLAST [53] for local chimera-checking and taxonomic assignment, respectively. All chimeric sequences were discarded. The pyrosequencing reads were deposited to the Short Read Archive database at NCBI (accession no. SRA061437).

### Statistical Analysis

Euclidean distances were calculated for 14 pore water and 13 sediment geochemical variables (Table 2) and used to construct a hierarchical cluster tree. Alpha diversity (within samples) including taxonomic richness (i.e., Chao1 and observed OTUs), Shannon diversity and equitability were calculated at the 80%, 90%, 95%, and 97% OTU levels [54], normalized by randomly re-sampling 958 sequences in each sample with 1000 replicates and any samples with <958 sequences were excluded from this analysis. Beta diversity (among samples) was also calculated. Weighted UniFrac distance [55] and Bray-Curtis dissimilarity were calculated at the 97% OTU level. The jackknifed unweighted pair group method with arithmetic mean (UPGMA) clustering was built from the calculated weighted UniFrac distances, and principal coordinates analysis (PCoA) and non-metric dimensional scaling (NMDS) were constructed from the Bray-Curtis dissimilarity to confirm if different ordinations would reveal the same pattern.

**Table 2:** Hot spring water and sediment physico-chemical properties.

In-situ sediment pore water physico-chemical parameters																		
Sample ID^a^		Temp	pH	TDS		F		Cl		NO_2_	Br	NO_3_	PO_4_	SO_4_	Ca	K	Mg	Na
		^o^C		mg/L														
GL13-4		66.0	7.8	2780		14.8		952.9		0.0	0.0	1.4	0.0	20.6	4.8	101.3	0.4	1141.5
GL20		43.0	7.4	2470		10.3		677.4		0.0	0.0	1.5	22.3	38.4	8.8	83.5	0.9	857.8
GL21		55.0	7.6	2380		11.1		708.8		0.0	0.0	1.8	25.3	42.5	12.3	96.3	2.5	871.2
GL28		75.0	7.9	2390		11.2		748.4		0.0	0.0	2.4	0.0	850.5	48.7	84.0	2.1	994.4
GL3-4		48.0	7.9	2060		11.0		797.7		0.0	0.0	4.7	20.9	34.0	7.7	145.0	1.9	1201.8
GL9		68.0	7.5	1404		7.3		410.1		0.0	0.0	0.6	0.0	44.4	2.1	54.8	0.1	489.9
GZ1		22.1	7.2	1560		7.9		145.7		11.4	11.6	16.1	22.7	140.0	1187.1	41.6	50.3	276.4
NM6		48.0	7.0	1069		0.0		141.1		0.0	2.6	76.2	0.0	174.0	2428.7	346.9	223.9	684.0
NM7		43.0	7.0	984		4.2		538.6		0.0	0.0	408.7	0.0	3.3	114.1	197.6	73.8	548.0
NQ4		49.0	7.8	398		1.5		72.7		0.6	0.0	40.0	0.0	31.7	40.9	62.2	49.4	264.9
QC2		75.0	7.6	1096		2.9		420.5		3.8	4.7	5.9	8.2	22.8	47.8	36.2	8.6	218.3
QC7		66.0	7.9	1123		2.5		385.3		0.0	0.0	1.0	0.0	14.1	38.9	39.6	8.1	247.0
QC9		60.0	8.1	1136		3.2		239.3		0.0	0.0	5.7	0.0	16.3	123.3	43.9	19.2	223.9
**Sediment TOC, TN and mineralogy**																		
	**GPS location(N/E)**				**TN**	**TOC**	**Illb**		**Kln**	**Sme**	**Cal**	**Kfs**	**Pl**	**Qtz**	**Dol**	**Ms**	**Py**	**Arg**
					**%**													
GL13-4	30°52′33.0″/91°36′38.8″				0.3	1.8	***24.0***		13.8	***21.6***	2.1	***17.4***	11.5	6.5	3.1	0.0	0.0	0.0
GL20	30°52′34.9″/91°36′34.0″				0.2	1.2	***19.4***		***22.6***	***21.9***	1.7	***17.1***	9.2	4.7	3.4	0.0	0.0	0.0
GL21	30°52′34.9″/91°36′34.0″				0.4	2.5	***20.5***		9.5	***40.8***	0.7	10.3	10.5	7.1	0.6	0.0	0.0	0.0
GL28	30°52′43.6″/91°36′50.6″				0.0	0.3	***15.0***		***17.1***	***27.1***	1.4	***19.7***	12.1	5.4	2.5	0.0	0.0	0.0
GL3-4	30°52′14.3″/91°36′48.7″				0.3	2.5	***23.6***		***20.4***	11.5	9.0	13.8	***17.1***	2.2	2.3	0.0	0.0	0.0
GL9	30°52′32.0″/91°36′37.8″				0.1	0.4	8.6		***15.6***	***33.2***	1.8	***22.5***	12.3	2.6	3.4	0.0	0.0	0.0
GZ1	31°40′52.6″/91°51′21.4″				0.1	0.3	13.5		6.8	11.7	***17.2***	***15.0***	7.6	***24.5***	*2.4*	*1.2*	*0.0*	*0.0*
NM6	31°44′34.1″/92°5′57.4″				0.3	9.7	3.1		4.6	***29.8***	***33.2***	7.7	6.1	1.6	4.5	2.5	1.3	5.5
NM7	31°44′33.6″/92°6′0.9″				0.3	10.3	***20.6***		9.3	12.0	***40.6***	6.3	5.2	3.0	2.8	0.0	0.1	0.0
NQ4	31°38′45.6″/91°45′8.2″				0.1	0.8	14.3		5.0	10.0	5.3	***18.2***	***16.2***	***23.8***	3.8	0.0	0.2	3.4
QC2	30°40′0.2″/91°35′28.9″				0.1	8.3	0.0		0.4	3.7	***24.7***	3.3	3.7	14.2	0.0	0.0	0.0	***49.8***
QC7	30°39′58.6″/91°35′28.6″				0.1	0.4	14.4		6.9	8.2	11.5	***16.9***	***37.9***	0.0	3.4	0.0	0.8	0.0
QC9	30°39′58.0″/91°35′29.4″				0.1	9.3	7.2		4.6	***24.2***	***36.9***	9.2	5.1	1.7	3.8	0.0	0.3	7.0
GLs^c^					0.1	0.6	12.5		***17.0***	***31.0***	1.7	***18.7***	14.4	4.1	0.8	0.0	0.0	0.0
NMs^c^					0.3	12.0	14.0		7.6	2.3	***48.2***	10.3	11.7	4.7	1.2	0.0	0.0	0.0

a Sample IDs were named according to the region in which a hot spring is located (such as GL) and the serial number of the spring (such as #13). In some cases, multiple samples were collected along a flow channel. For example, GL13.4 represents the 4th site along an outflow channel of the 13th hot spring in the Gulu area. Abbreviations: GZ: Guozu area; NM: Nima area; NQ: Naqu area; QC: Qucai area.

b The number in bold and italy are the major minerals (more than 15%) for each sample.

c GLs and NMs refer to soil samples from the Gulu and Nima area, respectively.

Analysis of similarity (ANOSIM) was performed to test for any significant difference in microbial community composition among different temperature ranges (moderate 75–66°C and low 60–22.1°C), or different sample types (hot spring sediments vs. adjacent soils). An R-statistic value of 1 (generated from ANOSIM) indicates distinct community composition between two groups. SIMPER (similarity percentage) analysis [56] was performed to rank the top ten taxa that contributed to the difference between any two groups. The average abundances of those taxa in each group were then calculated.

The BIO-ENV [57] procedure was used to reveal any correlation between community data (Bray-Curtis similarity) and scaled environmental data (Euclidean distance). The Envfit function was used to overlay the most significant environmental variables on the NMDS ordination. Mantel tests were also performed to check for any correlation between the whole community structure and specific environmental variables. All statistical analyses were performed in the QIIME pipeline and/or using the package Vegan in R [58].

## Results and Discussion

### Distinct Microbial Community Composition between Hot Spring Sediments and Adjacent Soils

**Figure 2:**
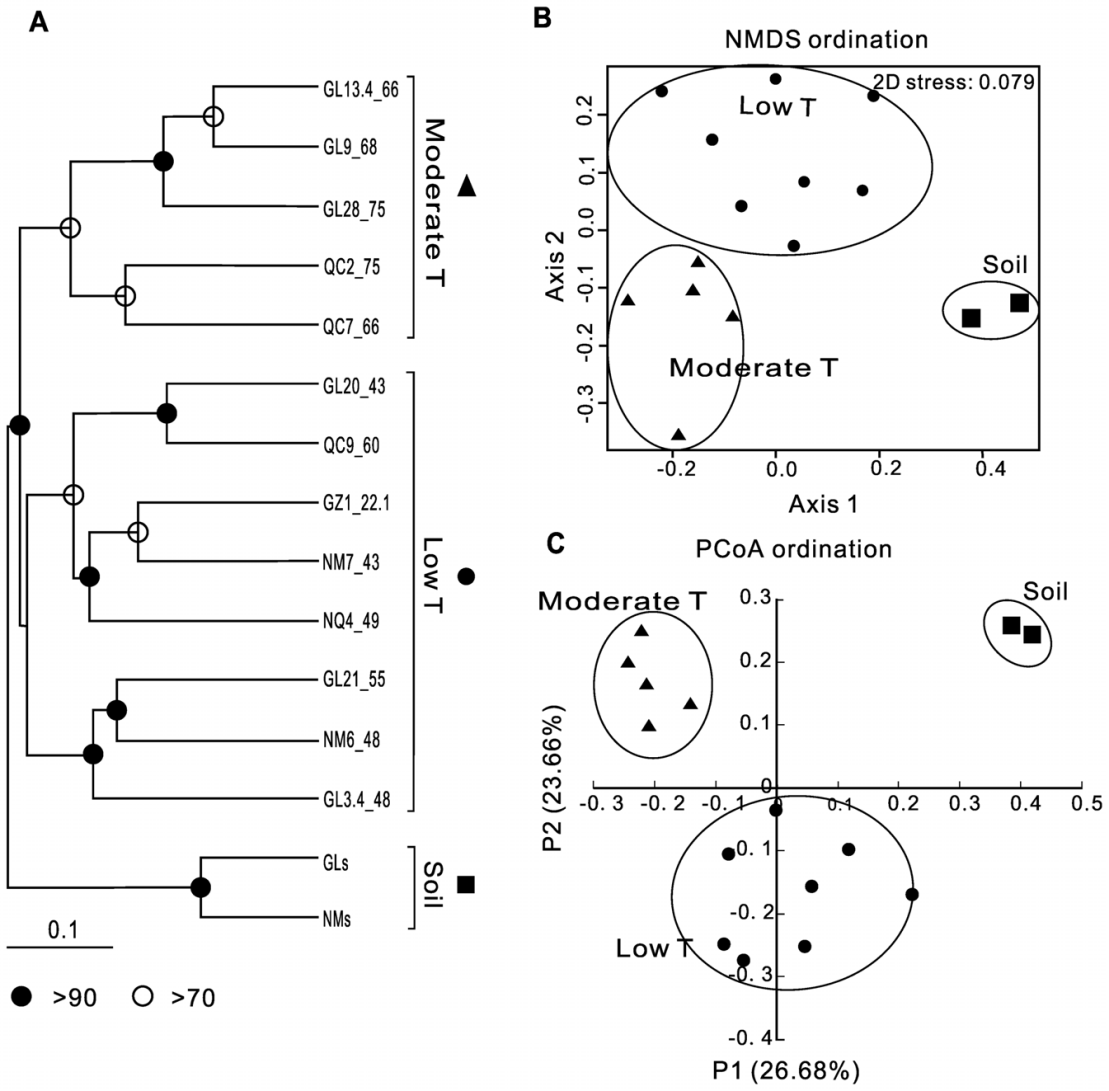
Microbial distribution pattern based on the complete pyrosequencing dataset at the 97% OTU level. **A.** Microbial cluster tree using jackknifed unweighted pair group method with arithmetic averages (UPGMA); **B.** Non-metric multidimensional scaling (NMDS) ordination for the community structure for all the samples. Ordination is based upon the Bray-Curtis similarity of the square-root-transformed abundances. The lower the 2D stress is, the better the ordination is; **C.** PCoA scatter plot that begins with a table of distances between the samples. The first two factors P1 and P2 can explain 26.7% and 23.7% variations, respectively. All three plots show the same pattern: the soil cluster, the low-temperature cluster, and the moderate-temperature cluster. The samples are primarily grouped by the sample type (hot spring sediments vs. adjacent soil); among the hot spring sediments, there are two groups separated by temperature (low vs. moderate temperature). Sample codes consist of sample ID and temperature for that spring. For example, GL13.4_66 represents the 4th site along an outflow channel of the 13th hot spring in the Gulu area with a temperature of 66°C. Different symbols are used to differentiate the different clusters.

**Figure 3:**
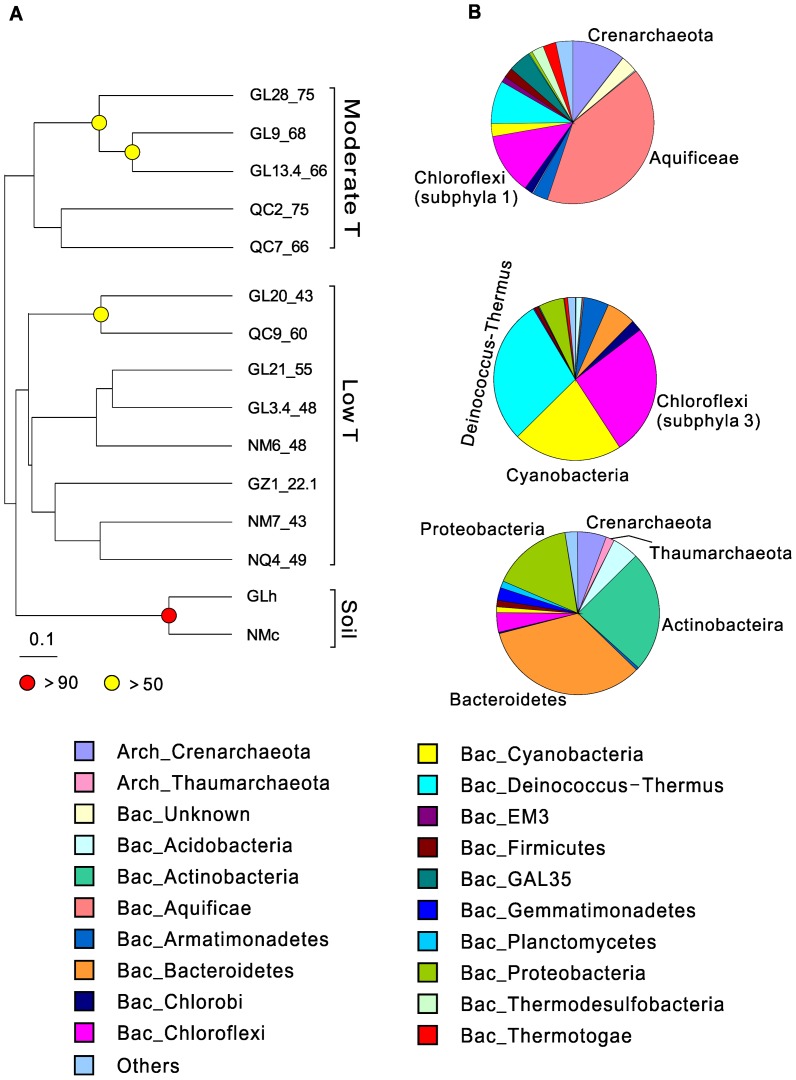
Comparison of microbial composition at the phylum-level OTUs among the three groups. The identified three groups are the soil cluster, the low-temperature cluster, and the moderate-temperature cluster**.** **A.** Cluster analysis using the complete dataset at the phylum-level OTUs; **B.** The pie charts show the relative proportions of microbial phyla in each group. Those phyla with <1% abundance (e.g. *Deferribacteres*, candidate phyla OP3, 8 and 9, SC4 and BRC1) are not shown and unknown bacteria are termed as “bac_other”. Dominant phyla marked on the pie charts are confirmed by the SIMPER analysis.

The qPCR results showed that *Bacteria* were more abundant than *Archaea* (10^8^–10^11^ copy/g vs. 10^7^–10^10^ copy/g). Cluster, PCoA, and NMDS (Figure 2A, 2B, and 2C respectively) analyses at the species-level OTUs (97%) revealed that all the samples were clustered into three distinct groups: moderate temperature springs (75–66°C), low temperature springs (60–22.1°C), and adjacent soils. Cluster analysis at the phylum-level OTUs (Figure 3A) revealed the same pattern. ANOSIM confirmed that these three groups were significantly different (ANOSIM R = 0.76, p = 0.001). The SIMPER procedure was used to identify the top ten OTUs responsible for the dissimilarity between the hot spring sediments and the soils (Table 3, the first panel). SCA1145 of Crenarchaea, *Arthrobacter* of Actinobacteria, and *Flavisolibacter* of Bacteroidetes were present in the soil samples only. In contrast, Aquificae (*Hydrogenobacter*), Cyanobacteria (*Cyanobacterium* and *Thermosynechococcus*), and Chloroflexi (*Roseiflexus* and *Oscillochloris*) were only observed in the hot spring sediments.

**Table 3:** SIMPER analysis identifies top ten taxon (at the 97% OTU level) that account for the most of the dissimilarities between the hot spring sediments and the adjacent soils, and between the moderate and the low temperature hot spring sediments.

Hot spring sediment vs. Soil					
Taxon^a^	Genus/Family^b^		Contr.(%)^c^	Avg^d^.hot spring (%)	Avg.soil (%)
Aquificae	*Hydrogenobacter*		4.9	18.2	0
Crenarchaeota	SCA1145		2.5	0	5.1
Actinobacteria	*Arthrobacter*		2.5	0	5.0
Cyanobacteria	*Cyanobacterium*		2.2	3.6	0
Chloroflexi	*Roseiflexus*		2.1	4.0	0
Cyanobacteria	*Thermosynechococcus*		1.7	10.0	0
Chloroflexi	*Oscillochloris*		1.6	2.7	0
Bacteroidetes	*Flavisolibacter*		1.5	0	3.5
GAL35	unidentified GAL35		1.3	3.8	0
**Moderate T vs. Low T**					
**Taxon**	**Genus/Family**		**Contr.(%)**	**Avg.moderate T(%)**	**Avg.low T(%)**
Aquificae	*Hydrogenobacter*		14.3	30.9	0.3
Deinococcus-Thermus	*Thermus*		9.1	7.5	23.9
GAL35	*unidentified GAL35*		3.8	4.3	0.1
Chloroflexi	*Roseiflexus*		3.7	0.0	5.2
Cyanobacteria	*Cyanobacterium*		3.7	0.0	4.9
Cyanobacteria	*Thermosynechococcus*		3.1	2.2	10.4
Chloroflexi	*Anaerolineae*		2.8	0	3.0
*Thermoprotei*	*Aeropyrum*		2.1	2.5	0.0
Chloroflexi	OPB11		1.9	2.0	0.0
*Thermoprotei*	*Aeropyrum*		1.8	2.0	0.0

a Phylum level for *Bacteria* and Class level for *Archaea*.

b In general, genus was displayed; if genus level was not available, family was displayed.

c Contribution of each OTU to the overall dissimilarity between the two clusters.

d Average abundance of each OTU.

**Figure 4:**
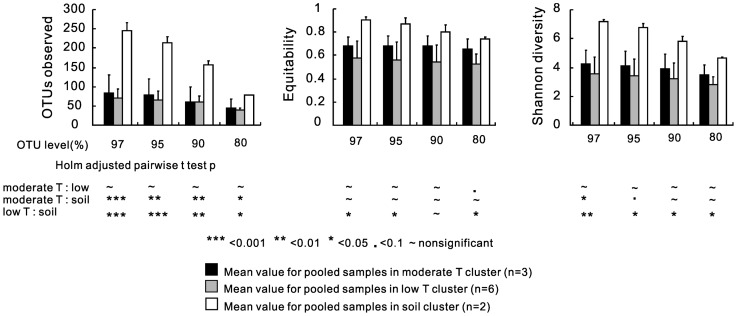
Comparison of alpha diversity. Calculation of microbial Richness, Equitability, and Shannon diversity among the pooled samples for the low temperature cluster (60–22.1°C), the moderate temperature cluster (75–66°C), and the soil cluster at four OTU levels: 97%, 95%, 90%, and 80%. Error bars indicate standard error of the mean. Pairwise t tests were performed for each pair of comparisons, moderate vs. low temperature, moderate temperature vs. soil, and low temperature vs. soil at each OTU level. Symbols ***indicates p<0.001; **p<0.01; *p<0.05;. p<0.1; ∼ non-significant. A Bonferrroni correction was made for the number of comparisons.

Alpha diversity revealed differences between the hot spring sediments and the soil samples (Figure 4). Pairwise t test showed that microbial diversity (observed OTUs and Shannon diversity) in the soil cluster was significantly higher than both the moderate and low temperature clusters at all defined OTU levels (Figure 4 and Table S1). Taxa in the soil cluster were distributed more evenly than those in the low temperature cluster at the OTU levels of 97%, 95%, and 80% (Figure 4, Table S1), which suggested that the soil communities may be more functionally stable than those in the low temperature springs [59].

Difference in microbial community composition between hot spring sediments and surrounding soils has been reported in other studies. A previous study of Tibetan hot springs [37] revealed that both archaeal and bacterial communities in soils were distinct from adjacent hot spring sediments as determined by LIBSHUFF clustering analysis. Another study [34] compared the relative abundance of crenarchaeol, a potential biomarker for AOA, in three crenarchaeol-rich hot springs and adjacent soils from the Great Basin of Nevada, USA, and concluded that soils were not significant sources of crenarchaeaol in the hot springs.

### Effect of Temperature on the Overall Microbial Composition and Diversity

Cluster and SIMPER analyses showed that temperature played an important role in controlling microbial community composition at the phylum (Figure 3B) and species levels (Table 3, the second panel). NMDS ordination at the species-level OTUs showed that the temperature vector pointed from low temperatures (60–22.1°C) to moderate temperatures (75–66°C) (Figure S1) with a high R-square value of 0.75 (p<0.05). Mantel test confirmed the importance of temperature to microbial community structure (r = 0.51, p = 0.001).

Distinct differences were observed in microbial composition between the moderate (75–66°C) and low temperature (60–22.1°C) groups (Figure 3, Table 3, the second panel). The majority of the sequences in the moderate temperature group (75–66°C) were affiliated with Aquificae (relative abundance was 40.8%), Chloroflexi (12.2%), Deinococcus-Thermus (8.9%), and GAL35 (4.3%). Some crenarchaeal sequences were also detected (10.4%) in this group. In the low temperature group (60–22.1°C), the microbial community was dominated by Deinococcus-Thermus (29.0%), Chloroflexi (26.0%), and Cyanobacteria (21.4%). SIMPER results (Table 3, the second panel) identified the top ten OTUs (at the 97% similarity level) that accounted for the dissimilarity in the community composition between the moderate (75–66°C) and low temperature (60–22.1°C) springs. *Hydrogenobacter*, GAL35, a novel bacterial lineage OPB11, and *Aeropyrum* mainly occurred in the moderate temperature (75–66°C) group, whereas *Roseiflexus*, *Cyanobacterium*, and *Anaerolineae* were only observed in the low temperature (60–22.1°C) springs. Other species, such as *Thermus* and *Thermosynechococcus* were more abundant in low temperatures than in moderate temperatures. Overall, these bacterial and archaeal communities are commonly observed in low-elevation springs and do not appear to be unique to high-elevation Tibetan springs.

The effect of temperature on hot spring microbial community composition has been reported by many studies [5], [7], [11], [14], [16], [28]. These studies investigated this effect by collecting samples either along a thermal gradient at various spatial scales [7], [11], [14] or across different springs with a wide temperature range [16], [28]. Temperature was found more important than biogeography in shaping microbial community composition. Some studies have shown that changes in microbial community composition were best explained by a combination of temperature and other environmental conditions, such as sulfide concentration [19] and pH [28]. In this study, we measured pore water chemistry (anions and cations) and sediment properties (mineralogy, TOC, and TN). Significant differences in pore water chemistry, mineralogy, TOC, and TN were observed among the springs (Table 2). Geochemical cluster analysis confirmed these differences among the springs (Figure S3). Interactions of minerals and microbes (Figure S2A) were also observed and were speculated to have an effect on microbial distribution [60]. However, the BIO-ENV analysis did not suggest that additional environmental factors, other than temperature, would improve the correlation between microbial community structure and environmental factors (Table S2). Furthermore, the relationship between community similarity and pairwise temperature difference between two springs showed a negative correlation (Figure S4), suggesting that microbial similarity decreased with increasing difference in temperature. This type of negative correlation has been previously observed [5], [14] and suggested that temperature exerted a strong control on microbial community composition.

Despite the distinct differences in microbial community structure between the moderate (75–66°C) and low temperature groups (60–22.1°C), the microbial richness and Shannon diversity at any OTU levels (80%, 90%, 95%, and 97%) were similar between these two groups (Figure 4, Table S1). Microbial richness/diversity has been observed to decrease with increased temperature for hot springs in Iceland [16], YNP [5], and Japan [11], but showed a positive linear correlation for hot springs in northern Thailand [19]. In contrast, this study and two other studies on Tibetan hot springs [26], [37] reached the same conclusion that microbial diversity did not show a monotonic relationship to temperature change.

### The Linear Relationship between the Relative Abundances of Specific Microbial Groups and Temperature

**Figure 5:**
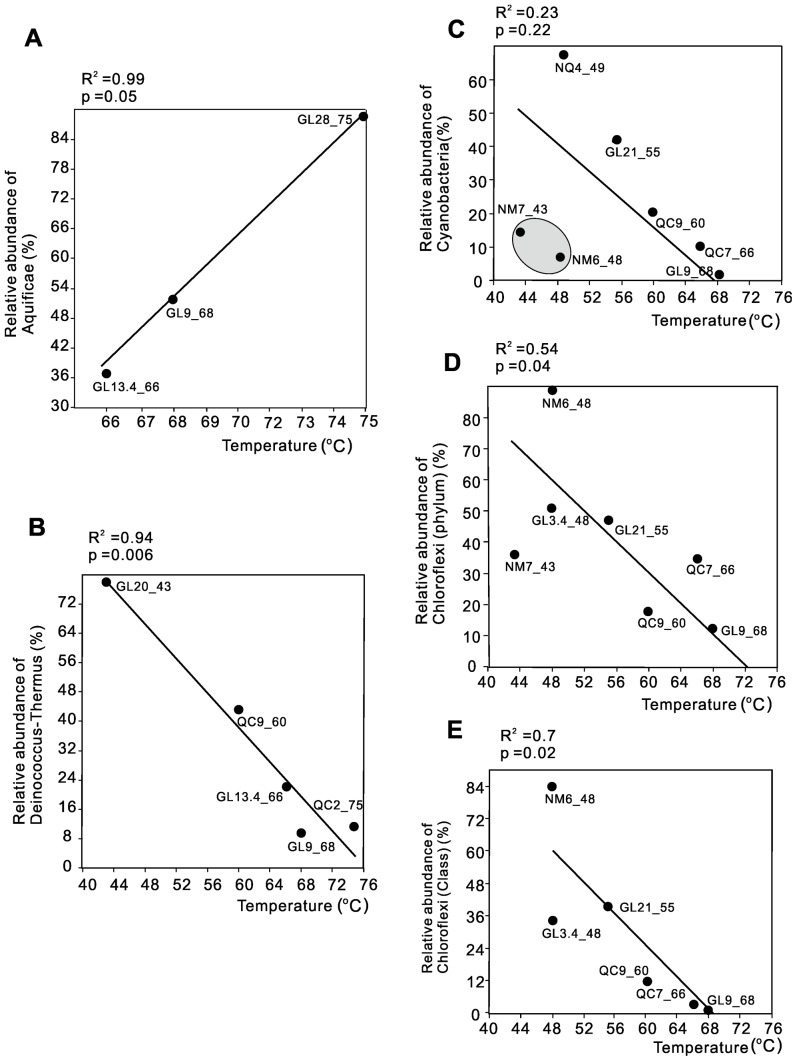
Correlations between the relative abundances of dominant microbial groups and temperature. The relative abundance of Aquificae (**A**) shows a positive correlation with temperature, whereas the relative abundances of the phyla Deinococcus-Thermus (**B**), Cyanobacteria (**C**) and Chloroflexi (**D**) as well as the class *Chloroflexi* (E) show negative correlations with temperature. The relative abundance of Cyanobacteria and temperature was not significantly correlated (R^2^ = 0.23; p<0.5) (Figure 5C); when the two outliers (springs NM6 and NM7, where Cyanobacteria were minor) were omitted, the linear correlation was dramatically improved (R^2^ = 0.90; p<0.005) (figure not shown). When qPCR data were used for Cyanobacteria (**C**) and class-level *Chloroflexi* (E), similar correlations resulted (data not shown for clarity).

Similar to the temperature-controlled variations in the overall microbial community composition, the relative abundances of various groups within *Bacteria* also varied with temperature change (Figure 5, Figure S5). Aquificae, mainly *Aquificales* occurred in high temperature springs and its relative abundance increased with temperature (Figure 5A, Figure S5A). The occurrence of *Aquificales* also appeared to be related to silica deposition. For example, the Gulu hot springs in the moderate temperature cluster hosted abundant *Aquificales* where siliceous sinters were observed in the field and spherules-like morphology of silica was observed under scanning electron microscope (SEM) (Figure S2B), as consistent with previous studies in YNP [1], [61] where *Aquificales* were often observed in silica-depositing springs. However, these observations are largely based on morphological evidence without knowing the exact mechanism of silica biomineralization. Certain laboratory experiments have provided some clues by showing that certain genera of *Aquificales* (e.g., *Sulfurihydrogenibium*) are able to extrude extracellular polymeric substances (EPS) to serve as nucleation sites for silica precipitation [62].

Within *Aquificales*, *Hydrogenobacter* was the predominant genus (Figure S5A) with a few sequences related to *Sulfurihydrogenibium* and *Balnearium* (first isolated from a black smoker chimney [63]). The relative proportions of these three genera varied as a function of temperature and other geochemical conditions. The abundance of *Hydrogenobacter* increased with temperature (Figure S5A), and in one spring, it co-existed with *Sulfurihydrogenibium*. The co-existence of these two genera was also observed in YNP hot springs under similar pH and temperature conditions (i.e., near neutral to slightly alkaline pH and a temperature range of 60–80°C). In YNP, *Hydrogenobacter* and *Sulfurihydrogenibium* usually co-exist in springs with high total sulfide concentrations (up to 12 mg/L) [27], but in Tibetan hot springs they appeared to co-exist in springs with low sulfide concentration (<0.1 mg/L) [31]. A previous study also showed the co-existence of these two genera in Thailand hot springs with low sulfide concentrations (0.16–2.85 mg/L) [19]. These studies collectively suggest that sulfide concentration is not a limiting factor for the co-existence of these two genera.

The presence of genus *Balnearium* within *Aquificales* in Tibetan hot springs was unexpected because it was recently recovered in a black smoker chimney [54], and was never reported in any terrestrial hot springs. Conversely, genus *Thermocrinis* was expected to be present in Tibetan hot springs because this genus was commonly found in other hot springs worldwide with near-neutral pH, high temperature (75–92°C), and low sulfide concentrations (usually <1 mg/L) [1], [8], [16], [64], but it was completely absent in Tibetan springs. The possible reason included low temperatures of Tibetan springs relative to the growth temperature range (75–92°C) of this genus. However *Thermocrinis* was not observed in Tengchong hot springs either, where spring temperatures were suitable for its growth [28]. Therefore, other reasons, such as biogeography and endemism, may be important as well.

The phylum Deinococcus-Thermus in Tibetan hot springs was dominated by *Thermus* with some *Meiothermus* and *Deinococcus* (Figure S5B). The sum of *Thermus* and *Meiothermus* contributed to the negative correlation between the abundance of Deinococcus-Thermus and temperature (Figure 5B). The temperature response of the genera *Thermus* and *Meiothermus* was consistent with their respective physiologies. Most species of *Thermus* can grow anaerobically in the presence of nitrate with an optimal growth temperature of 65–75°C [65], whereas *Meiothermus* spp. have a lower optimal growth temperature 50–65°C with a maximal temperature at <70°C and exhibit O_2_ respiration [65]. Genera *Thermus* and *Meiothermus* have been common in global hot springs with moderate-high temperatures of 50–99°C and slightly acidic to alkaline pH, such as in Iceland [16], Kamchatka in Russia [22], Long Valley Caldera [66], YNP [67] and the Great Basin [12] of the United States, and Thailand [19]. However, *Thermus* and *Meiothermus* are usually not dominant in terrestrial hot springs, except for a few springs in Iceland [16]. The dominance of these two genera in low temperature Tibetan hot springs can be partially explained by high organic carbon content (Table 2), because *Thermus* isolates from hot springs are usually chemoorganotrophic [65] and can actively out-compete other organotrophs, such as *Anaerolinea* spp. [68].

The relative abundances of Cyanobacteria and Chloroflexi exhibited a negative correlation with temperature (Figure 5C and D, Figure S5C and S5D). The linear correlation between the Cyanobacteria abundance and temperature was dramatically improved (R^2^ = 0.90; p<0.005) after two outliers (springs NM6 and NM7) were omitted. Within these two outlier springs, Cyanobacteria were scarce but Chloroflexi were abundant. Five genera constituted the majority of the cyanobacterial sequences and they inhabited springs with different temperature ranges (Figure S5C). At moderate temperatures (75–55°C), sequences related to *Synechococcales* (e.g., *Synechococcus* and *Thermosynechococcus*) were abundant, consistent with the results of two early studies on Tibetan hot springs (62–70°C) [26], [29]. At lower temperatures, *Chroococcales* (e.g., *Cyanobacterium*) and *Pseudanabaenales*, were more abundant.

The sub-phylum classification of Chloroflexi adopted in this study followed a previous recommendation [68] and almost all the sub-phyla (classes) of Chloroflexi were observed in Tibetan springs (Figure S5D). Sub-phylum 1 (*Anaerolineae*) and Sub-phylum 3 (*Chloroflexi*) were ubiquitous in Tibetan springs (Figure S5D). Sub-phylum 1 includes classes *Anaerolineae* and *Caldilineae* which are chemoorganotrophs: the former is an anaerobic fermenter producing acetate and H_2_ as well as lactate when glucose and yeast extract are present; the latter obtains energy from O_2_ respiration [69]. The sum of all three genera within sub-phylum 3, *Oscillochloris*, *Roseiflexus*, and *Chloroflexus*, showed a negative correlation with temperature (Figure 5E and Figure S5 inset) and occurred as long filaments under SEM (Figure S2C).

### Inferred Correlation between Cyanobacteria and Filamentous Anoxygenic Phototrophic Chloroflexi

**Figure 6:**
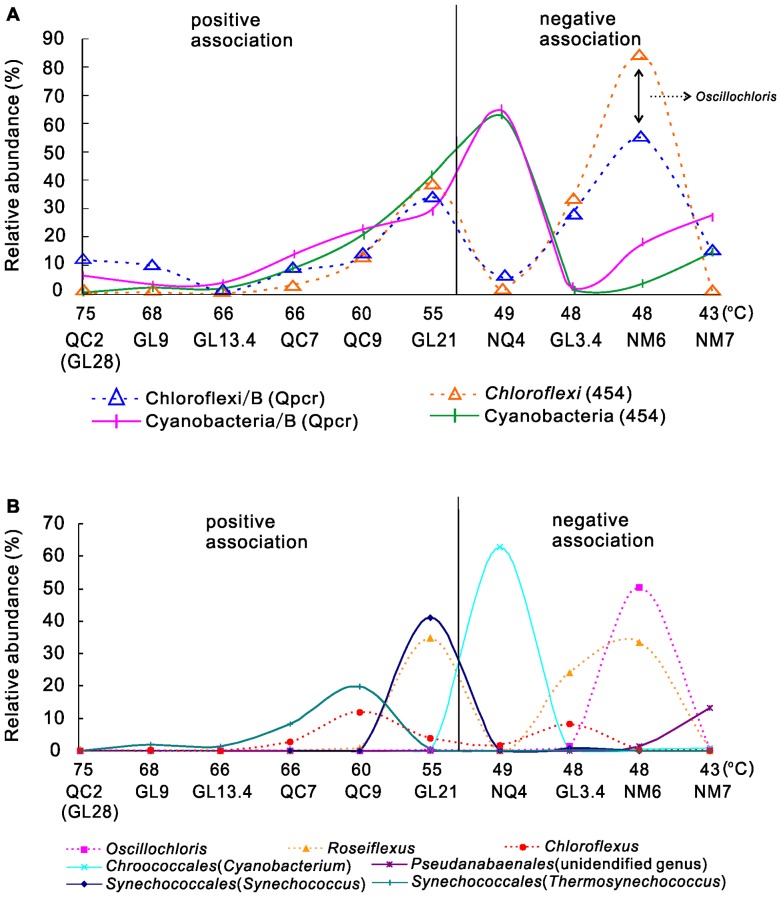
Distribution of major Cyanobacteria and Chloroflexi genera across a wide temperature range. The solid lines represent Cyanobacteria and the dash lines for Chloroflexi. **A.** The relative abundance of total Cyanobacteria and Filamentous Anoxygenic Phototrophic Chloroflexi (FAPs) as determined by qPCR and 454 pyrosequencing across a wide temperature range. Both methods showed a similar trend: in the range of 75–55°C, the abundance of Cyanobacteria was positively correlated with that of FAPs; in the range of 55–43°C, the abundance of Cyanobacteria was negatively correlated with that of FAPs. The difference in the relative abundance of Chloroflexi between the qPCR and the 454 results for Spring NM6 (Figure 6) was likely caused by different primers used: the 454 primer for Chloroflexi included *Oscillochloris*, but the qPCR primer did not include this genus. **B.** The relative abundance of three genera within FAPs and major genera within Cyanobacteria as a function of temperature. Each genus has its optimal temperature. The sum of these genera within FAPs and Cyanobacteria contributed to the positive or negative correlations between these two phyla at different temperature ranges. For Cyanobacteria, those organisms with the relative abundance of >5% were included, so *Planktothricoides* in Figure S5C were not included in Figure 6.

Cyanobacteria and filamentous anoxygenic phototrophs (FAPs, namely organisms belonging to the order *Chloroflexales*) are ubiquitous mat-forming phototrophs in neutral to alkaline hot springs (reviewed in [70], [45]). The difference is that the former group includes oxygenic phototrophs, whereas the latter (i.e., *Chloroflexus, Oscillochloris,* and *Roseiflexus*) includes mixtrophs [45] that are capable of carrying out photosynthesis without producing oxygen. In general, FAPs such as *Chloroflexus* and *Oscillochloris* grow best photoheterotrophically; however, photoautotrophy can be an alternative pathway to support their metabolism, especially when sulfide concentration is <1 mg/L [41]–[43] and light intensity is low [45]. Therefore, Cyanobacteria and FAPs may not always be a cooperative relationship (i.e., a producer-consumer relationship) [71], but instead they may compete against each other for limiting nutrients [5]. Our qPCR results and the relative abundance data consistently supported this complex interaction pattern between Cyanobacteria and filamentous *Chloroflexi* (Figure 6). Within the temperature range of 75–55°C (including 55°C), a positive correlation was observed between Cyanobacteria and FAPs (r = 0.99, p<0.001) (Figure 6A), which suggested that FAPs (*Chloroflexus*, *Oscillochloris*, and *Roseiflexus*) could be heterotrophic and may be dependent on organic carbon synthesized by Cyanobacteria for their growth. However, within 55–43°C the abundance of Cyanobacteria was negatively correlated with that of FAPs (r = −0.60, p<0.05) (Figure 6A), which suggested that FAPs may become photoautotrophic and compete against Cyanobacteria for available nutrients and/or physical space. This competitive relationship has been previously reported in YNP hot springs [5] and has been demonstrated by additional investigations on the photosynthetic activity of oxygenic and anoxygenic phototrophs in a hypersaline microbial mat, where anoxygenic phototrophs (*Chloroflexus*-like bacteria) outcompeted oxygenic phototrophs (Cyanobacteria) for inorganic carbon with full-light illumination [44]. A close examination revealed changes in genus composition within Cyanobacteria and FAPs as a function of temperature (Figure 6B). Therefore, the temperature-dependent relationship between Cyanobacteria and FAPs was likely due to differential adaptations of various cyanobacterial and FAP genera to temperature change. Whereas all genera of Cyanobacteria are photoautotrophic, those within FAPs can be either autotrophic or heterotrophic. Despite these encouraging results, definitive evidence for the complex relationship between Cyanobacteria and FAPs must await further research, such as rate measurements using labeled ^14^CO_2_.

### Higher Microbial Diversity Observed than Previous Tibetan Hot Spring Studies

Previous studies have revealed fairly high biodiversity in Tibetan hot springs [26], [29], [37], however our pyrosequencing-based results revealed even higher richness. For *Bacteria*, the number of observed OTUs at the 97% similarity level (corresponding to the species level) in the low temperature cluster was approximately 5-fold higher than the highest number of OTUs observed in two previous Tibetan hot spring studies [26], [37]. A total of 42 bacterial phyla were obtained at 97% OTU level in our study, in comparison with only 10 observed previously [26], [37]. The major bacterial phyla detected in our study varied systematically with temperature (Figure 3 and 5). In contrast, the dominant bacterial phyla identified in a recent study [37] including Firmicutes, Proteobacteria and Cyanobacteria/Chloroflexi did not exhibit any particular distribution pattern within a temperature range of 26–81°C. Another major difference observed between this study and previous studies was the occurrence of the hyperthermophiles Aquificae: in this study, Aquificae was the dominant phylum in moderate temperature springs, whereas in previous Tibetan hot spring studies either a small fraction of Aquificae (3 sequences, 6.7%) [37] or no Aquificae sequences [26] were obtained.

These differences between our pyrosequencing-based results and previous 16S rRNA clone library based studies [26], [37] may be caused by two reasons: first, different sequencing methods were used. For example, pyrosequencing can retrieve minor microorganisms and achieve higher sensitivity than the 16S rRNA clone library approach [72]; second, spatial and temporal variations in Tibetan hot springs. One early study [26] sampled the springs in the Daggyai Tso geothermal region of the southern central Tibet in 2003, while samples used in a more recent study [37] and our study came from the central-eastern Tibet, which were collected in 2009 and 2010, respectively. Even in the same sampling area, different springs could host different communities, because local physicochemical conditions are important factors in shaping community structure. Therefore, it is reasonable to observe spatial and temporal differences in bacterial community composition.

### Conclusions

Microbial community structure and diversity did not show any location-specific variations. Instead, microbial community composition varied systematically with temperature: moderate temperatures (75–66°C) favored Aquificae, *Archaea*, and GAL35, whereas when temperature decreased to 60–22.1°C, Deinococcus-Thermus, Cyanobacteria and Chloroflexi became dominant microorganisms. At the genus level, microorganisms commonly observed in other lower-elevation terrestrial hot springs also occurred in high-elevation Tibetan hot springs, such as *Hydrogenobacter* spp. and *Sulfurihydrogenibium* spp. within Aquificae, *Thermus* spp. and *Meiothermus* spp. within Deinococcus-Thermus, *Synechococcus* spp., *Thermosynechococcus* spp. and *Cyanobacterium* spp. within Cyanobacteria, *Oscillochloris* sp., *Roseiflexus* spp. and *Chloroflexus* spp. within Chloroflexi. In addition, the relative abundance of Aquificae was positively correlated to temperature, but the opposite was true for the phyla Deinococcus-Thermus, Cyanobacteria and Chloroflexi, as well as the class Chloroflexi. The relationship between Cyanobacteria and filamentous *Chloroflexi* was positive at low temperatures (55–43°C), but negative at higher temperatures (75–55°C). This complex relationship between Cyanobacteria and FAPs was likely due to multiple environmental factors, among which temperature could be an important one.

## Supporting Information

Figure S1 **Temperature fitted NMDS ordination.** NMDS ordination was analyzed with the complete 454 dataset at the 97% OTU level. This figure shows that microbial community structure is structured primarily according to temperature.
(TIF)

Figure S2 **SEM photographs showing microorganisms and minerals in Tibetan hot spring sediments.** **A.** A SEM photograph showing associations of microbes and clay minerals (plate-like morphology) and silica (spherules-like morphology). Identification of these minerals was based on a combination of XRD and energy dispersive spectroscopy (EDS) (data not shown). **B.** A SEM photograph for sample GL28 (75°C) showing abundant Aquificae (filaments) in association with silica (spherules-like morphology). The identification of Aquificae was based on its morphology and the fact that Aquificae accounted for 90% of all prokaryotes in this sample; **C.** A SEM photograph for the GL3.4 sediment (48°C) where abundant filamentous *Chloroflexi* were observed (black arrows). Again the identification of filamentous *Chloroflexi* was based on its morphology and that fact that filamentous *Chloroflexi* constituted ∼35% of total prokaryotes.
(DOC)

Figure S3 **A hierarchical tree for pore water and sediment geochemistry** **based on Euclidean distances.** This figure shows a water and sediment geochemistry clustering pattern primarily according to the geographic location.
(TIF)

Figure S4 **Decay dynamics of community similarity with increased difference in temperature.** Y-axis represents the community similarity using Unifrac distance; X-axis represents the pair-wise temperature difference between a pair of spring samples.
(TIF)

Figure S5 **The relationship between the relative abundance of various groups and temperature in Tibetan hot springs.** **A.** The genera of Aquificae observed in this study; **B.** The genera of Deinococcus-Thermus; **C.** Cyanobacterial orders and genera **D.** Sub-phyla (class level) of the phylum Chloroflexi and genera within Sub-phylum3 (filamentous *Chloroflexi*).
(TIF)

Table S1 **Alpha-diversity indices based on the entire 454 dataset at the 97% OTU similarity level.** This table only contains those samples with sequence number >958.
(DOC)

Table S2 **The BIO-ENV results.** This table shows various correlations between microbial community composition (based on the 97% OTU level) and a subset of environmental variables. All the environmental variables are from Table 2.
(DOC)
