# Correction: Control of Temperature on Microbial Community Structure in Hot Springs of the Tibetan Plateau

**DOI:** 10.1371/journal.pone.0099751

**Published:** 2014-06-02

**Authors:** The PLOS ONE Staff

The penultimate sentence of the Conclusions section is incorrect. The correct sentence is:

“The relationship between Cyanobacteria and filamentous *Chloroflexi* was negative at low temperatures (55–43°C), but positive at higher temperatures (75–55°C).”
