# Supplementary figures and images for: Control of Temperature on Microbial Community Structure in Hot Springs of the Tibetan Plateau
Source: PLoS One. 2013 May 7;8(5):e62901. doi: 10.1371/journal.pone.0062901 (PMC3647046; doi:10.1371/journal.pone.0062901)

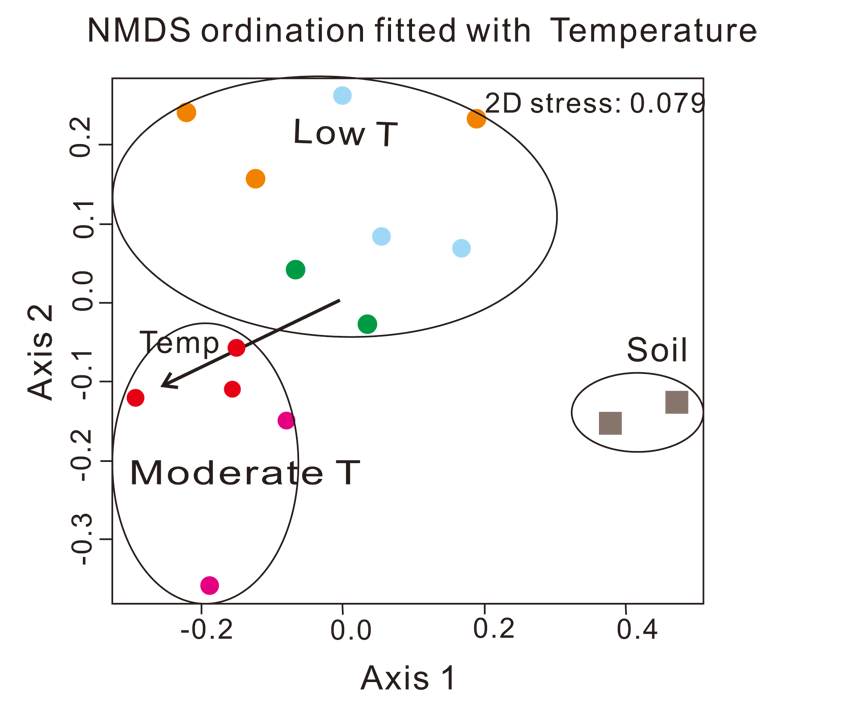

Supplement: Figure S1 — Temperature fitted NMDS ordination. NMDS ordination was analyzed with the complete 454 dataset at the 97% OTU level. This figure shows that microbial community structure is structured primarily according to temperature. (TIF) [file pone.0062901.s001.tif]

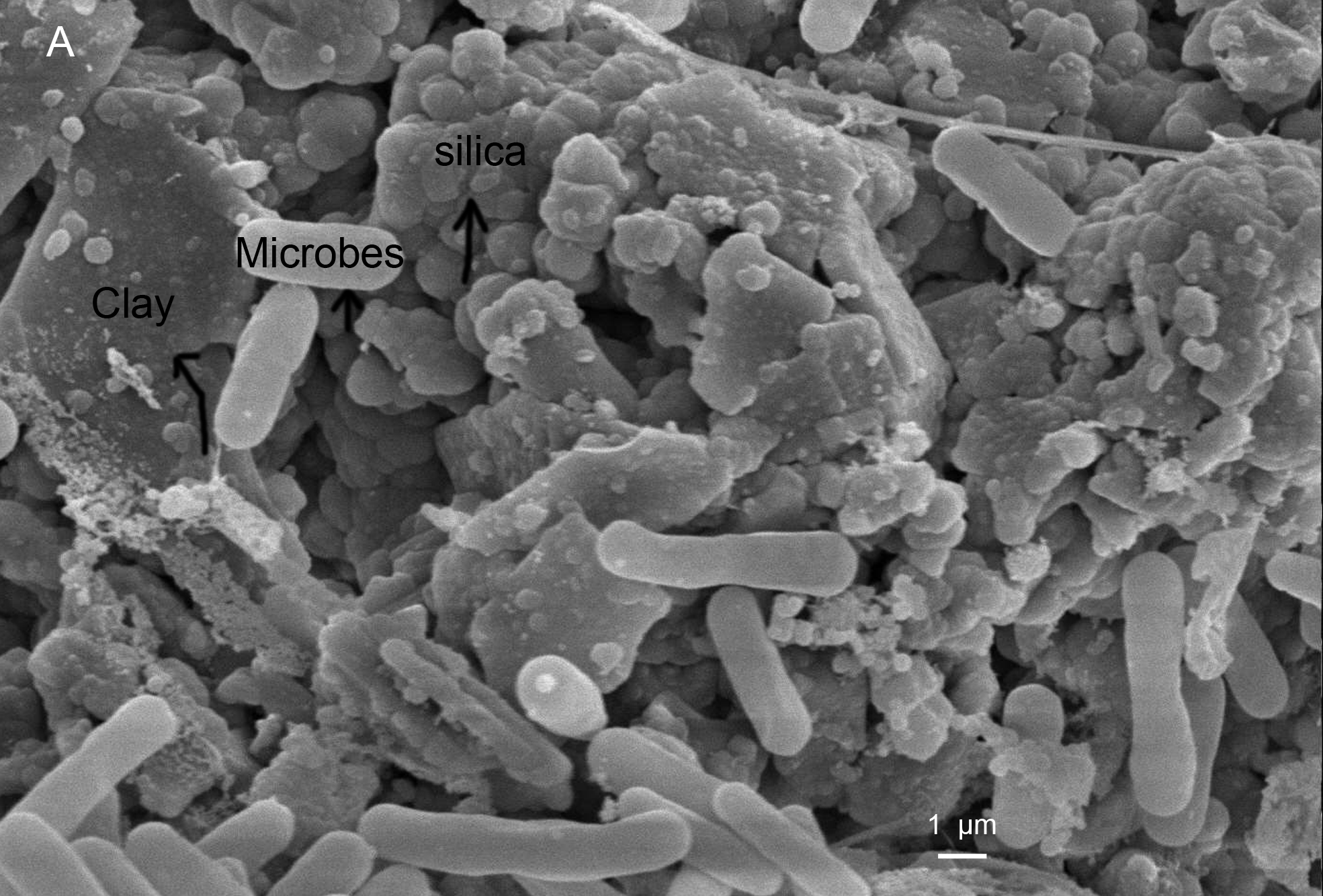


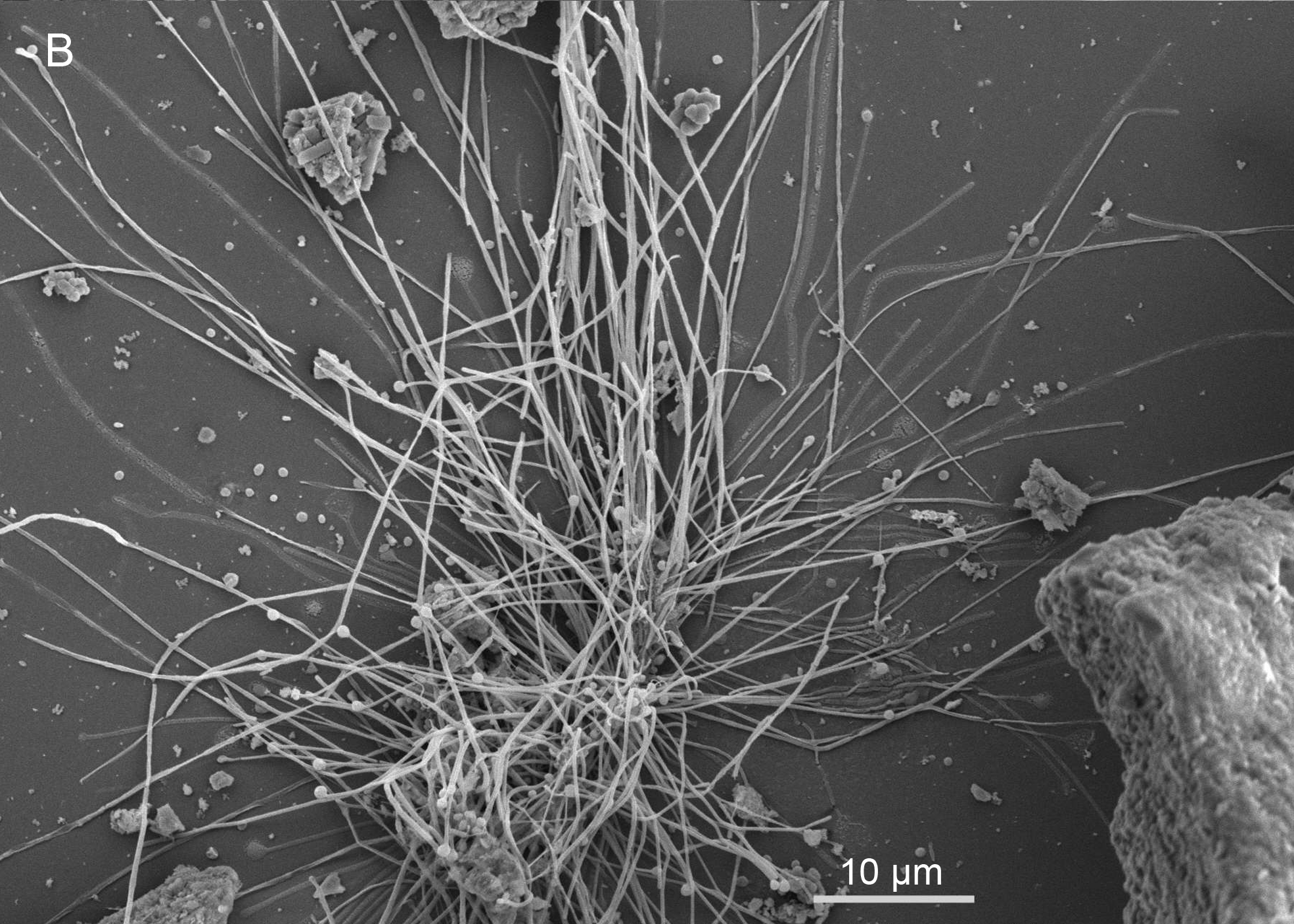

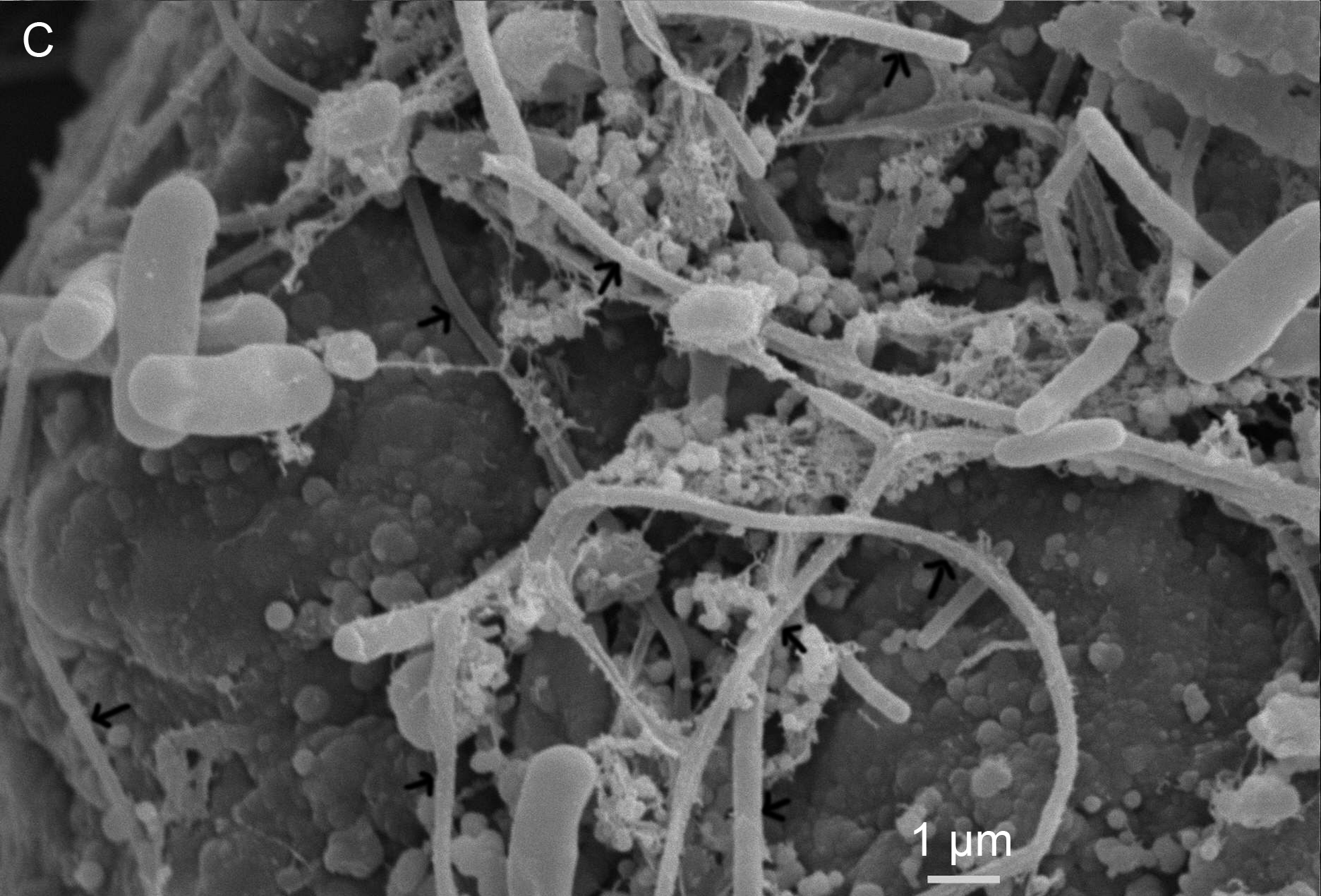

Supplement: Figure S2 — SEM photographs showing microorganisms and minerals in Tibetan hot spring sediments. A. A SEM photograph showing associations of microbes and clay minerals (plate-like morphology) and silica (spherules-like morphology). Identification of these minerals was based on a combination of XRD and energy dispersive spectroscopy (EDS) (data not shown). B. A SEM photograph for sample GL28 (75°C) showing abundant Aquificae (filaments) in association with silica (spherules-like morphology). The identification of Aquificae was based on its morphology and the fact that Aquificae accounted for 90% of all prokaryotes in this sample; C. A SEM photograph for the GL3.4 sediment (48°C) where abundant filamentous Chloroflexi were observed (black arrows). Again the identification of filamentous Chloroflexi was based on its morphology and that fact that filamentous Chloroflexi constituted ∼35% of total prokaryotes. (DOC) [file pone.0062901.s002.doc]

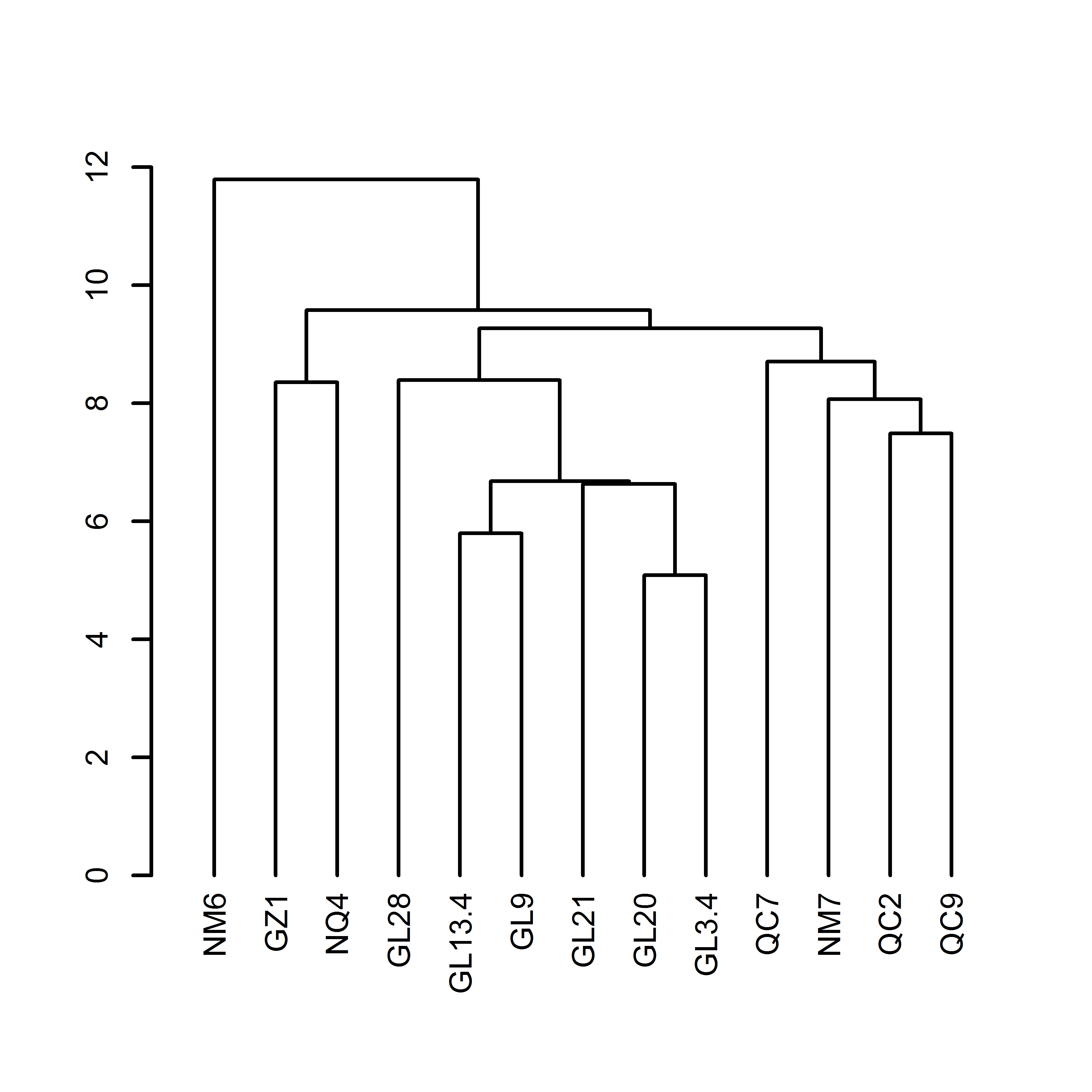

Supplement: Figure S3 — A hierarchical tree for pore water and sediment geochemistry based on Euclidean distances. This figure shows a water and sediment geochemistry clustering pattern primarily according to the geographic location. (TIF) [file pone.0062901.s003.tif]

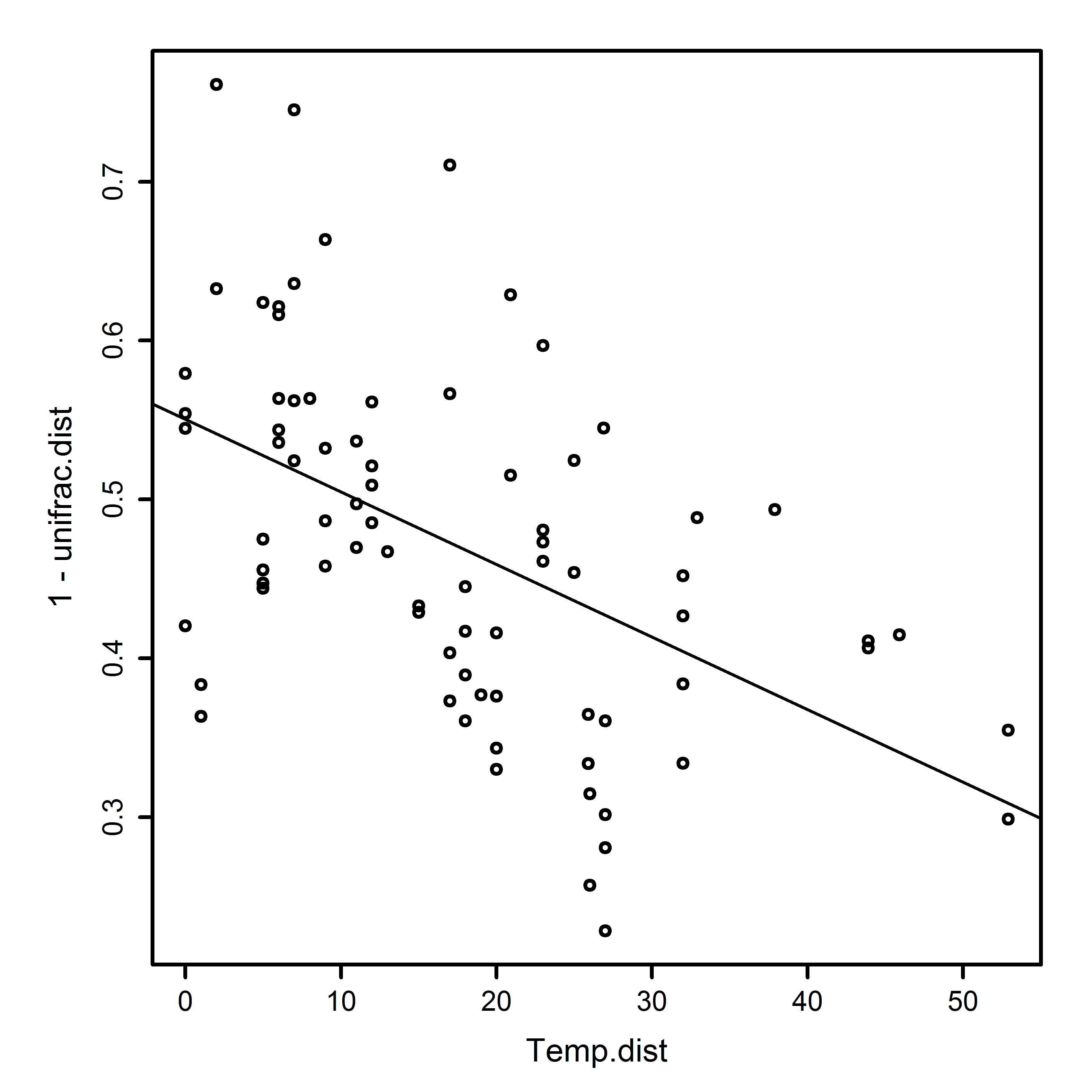

Supplement: Figure S4 — Decay dynamics of community similarity with increased difference in temperature. Y-axis represents the community similarity using Unifrac distance; X-axis represents the pair-wise temperature difference between a pair of spring samples. (TIF) [file pone.0062901.s004.tif]

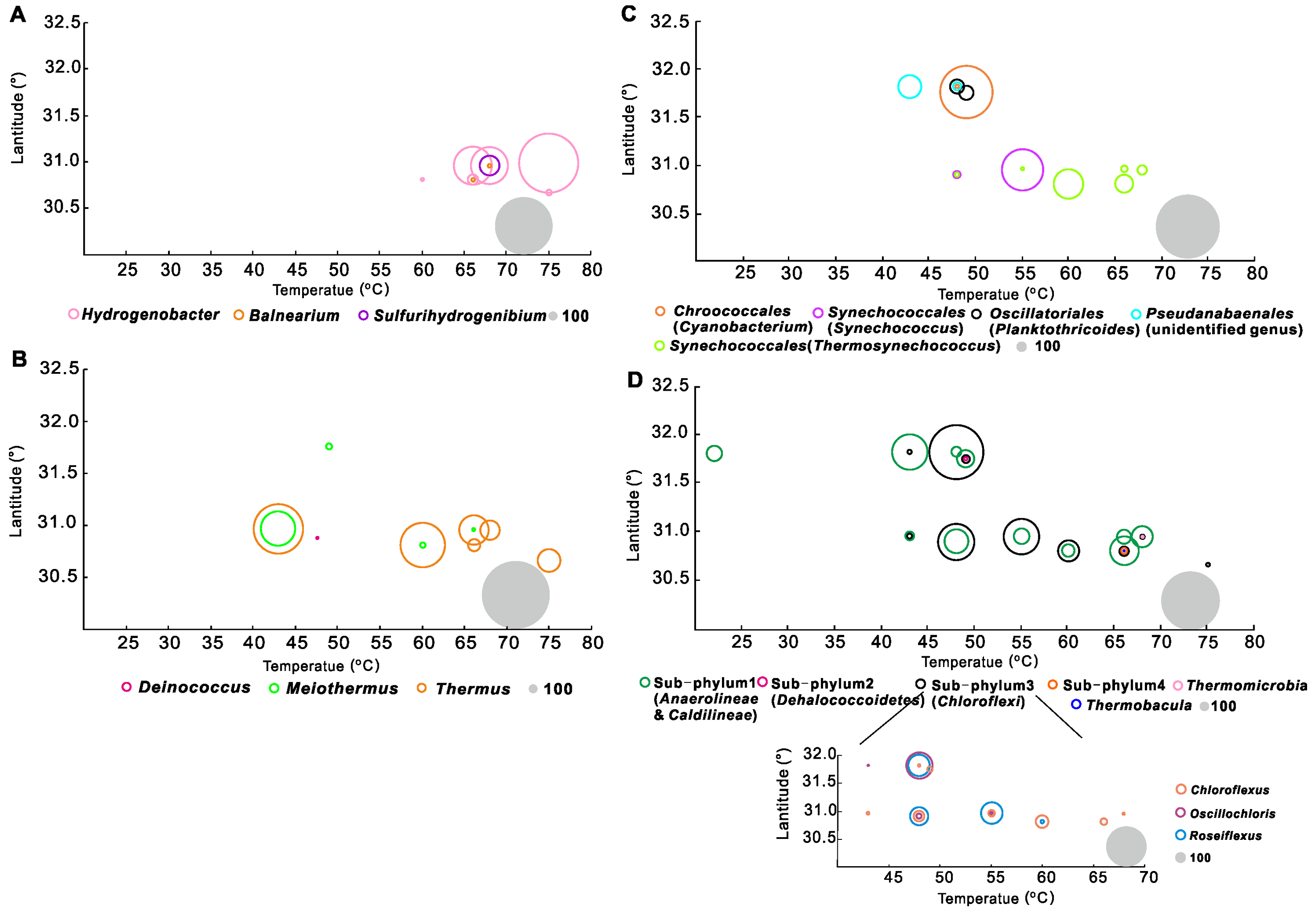

Supplement: Figure S5 — The relationship between the relative abundance of various groups and temperature in Tibetan hot springs. A. The genera of Aquificae observed in this study; B. The genera of Deinococcus-Thermus; C. Cyanobacterial orders and genera D. Sub-phyla (class level) of the phylum Chloroflexi and genera within Sub-phylum3 (filamentous Chloroflexi). (TIF) [file pone.0062901.s005.tif]
