# Supplementary material for: Control of Temperature on Microbial Community Structure in Hot Springs of the Tibetan Plateau
Source: PLoS One. 2013 May 7;8(5):e62901. doi: 10.1371/journal.pone.0062901 (PMC3647046; doi:10.1371/journal.pone.0062901)
Supplement: Table S1 — Alpha-diversity indices based on the entire 454 dataset at the 97% OTU similarity level. This table only contains those samples with sequence number >958. (DOC) [file pone.0062901.s006.doc]

**Table S1** Alpha-diversity indices; this table only contained samples with sequences > 958

|  | OTU level | GL9_68 | QC2_75 | QC7_66 | GL20_43 | QC9_60 | NM7_43 | NQ4_49 | GL3.4_48 | NM6_48 | GLh | NMc |
| --- | --- | --- | --- | --- | --- | --- | --- | --- | --- | --- | --- | --- |
| Chao1 | 97% | 162 | 51 | 159 | 75 | 145 | 108 | 66 | 108 | 51 | 351 | 259 |
|  | 95% | 128 | 45 | 145 | 66 | 121 | 104 | 60 | 103 | 46 | 299 | 228 |
|  | 90% | 85 | 30 | 117 | 76 | 89 | 89 | 70 | 96 | 42 | 220 | 184 |
|  | 80% | 58 | 25 | 90 | 48 | 59 | 54 | 42 | 69 | 49 | 98 | 85 |
|  |  |  |  |  |  |  |  |  |  |  |  |  |
| observed OTUs | 97% | 98 | 35 | 121 | 58 | 91 | 99 | 55 | 82 | 46 | 259 | 229 |
|  | 95% | 85 | 31 | 116 | 55 | 80 | 97 | 50 | 77 | 39 | 226 | 202 |
|  | 90% | 63 | 22 | 97 | 52 | 60 | 83 | 54 | 70 | 36 | 163 | 149 |
|  | 80% | 44 | 20 | 68 | 33 | 41 | 41 | 35 | 50 | 35 | 79 | 77 |
|  |  |  |  |  |  |  |  |  |  |  |  |  |
| Equitability | 97% | 0.60 | 0.67 | 0.76 | 0.46 | 0.57 | 0.81 | 0.49 | 0.70 | 0.45 | 0.88 | 0.93 |
|  | 95% | 0.59 | 0.68 | 0.76 | 0.46 | 0.56 | 0.80 | 0.48 | 0.66 | 0.39 | 0.84 | 0.91 |
|  | 90% | 0.59 | 0.68 | 0.77 | 0.44 | 0.55 | 0.77 | 0.45 | 0.65 | 0.39 | 0.76 | 0.84 |
|  | 80% | 0.56 | 0.71 | 0.71 | 0.40 | 0.53 | 0.61 | 0.51 | 0.62 | 0.48 | 0.74 | 0.75 |
|  |  |  |  |  |  |  |  |  |  |  |  |  |
| Shannon diversity | 97% | 3.95 | 3.45 | 5.28 | 2.71 | 3.69 | 5.37 | 2.82 | 4.42 | 2.50 | 7.05 | 7.25 |
|  | 95% | 3.78 | 3.37 | 5.24 | 2.65 | 3.54 | 5.31 | 2.72 | 4.16 | 2.08 | 6.59 | 6.94 |
|  | 90% | 3.54 | 3.03 | 5.05 | 2.53 | 3.22 | 4.90 | 2.61 | 4.02 | 2.01 | 5.56 | 6.08 |
|  | 80% | 3.04 | 3.03 | 4.31 | 2.02 | 2.85 | 3.27 | 2.62 | 3.52 | 2.43 | 4.64 | 4.72 |
