# Supplementary material for: Control of Temperature on Microbial Community Structure in Hot Springs of the Tibetan Plateau
Source: PLoS One. 2013 May 7;8(5):e62901. doi: 10.1371/journal.pone.0062901 (PMC3647046; doi:10.1371/journal.pone.0062901)
Supplement: Table S2 — The BIO-ENV results. This table shows various correlations between microbial community composition (based on the 97% OTU level) and a subset of environmental variables. All the environmental variables are from Table 2. (DOC) [file pone.0062901.s007.doc]

**Table S2** BIO-ENV results showing correlation between microbial community composition and each subset of environmental variables

|  | size | Correlation coefficient |
| --- | --- | --- |
| Temp | 1 | 0.6862 |
| Temp NO_3_ | 2 | 0.616 |
| Temp NO_2_ NO_3_ | 3 | 0.5966 |
| Temp TN NO_2_ NO_3_ | 4 | 0.5967 |
| Temp NO_2_ NO_3_ SO_4_ Mg | 5 | 0.6114 |
| Temp TN NO_2_ NO_3_ SO_4_ Mg | 6 | 0.6173 |
| Temp TDS TN NO_2_ NO_3_ SO_4_ Mg | 7 | 0.615 |
| Temp TDS TN F NO_2_ NO_3_ SO_4_ K | 8 | 0.6069 |
| Temp TN F NO_2_ NO_3_ PO_4_ SO_4_ K Na | 9 | 0.6035 |
| Temp TN Cl NO_2_ Br NO_3_ PO_4_ SO_4_ K Na | 10 | 0.5898 |
| Temp pH TDS TN Cl NO_2_ NO_3_ PO_4_ SO_4_ K Na | 11 | 0.5591 |
| Temp pH TDS TN Cl NO_2_ Br NO_3_ PO_4_ SO_4_ K Na | 12 | 0.5498 |
| Temp pH TDS TN TOC Cl NO_2_ Br NO_3_ PO_4_ SO_4_ K Na | 13 | 0.5267 |
| Temp pH TDS TN TOC F NO_2_ Br NO_3_ PO_4_ SO_4_ K Mg Na | 14 | 0.4821 |
| Temp pH TDS TN TOC Cl NO_2_ Br NO_3_ PO_4_ SO_4_ Ca K Mg Na | 15 | 0.46 |
| Temp pH TDS TN TOC F Cl NO_2_ Br NO_3_ PO_4_ SO_4_ Ca K Mg Na | 16 | 0.4345 |
